# To be, or not to be, part-time in academia

**DOI:** 10.7554/eLife.106336

**Published:** 2025-02-20

**Authors:** Sinead English, M Emília Santos, Clare Buckley, Chrissy L Hammond, Sarah Lloyd-Fox, Nina F Ockendon-Powell

**Affiliations:** 1 School of Biological Sciences, University of Bristol Bristol United Kingdom; 2 Department of Zoology, University of Cambridge Cambridge United Kingdom; 3 Division of Molecular and Cellular Function, University of Manchester Manchester United Kingdom; 4 School of Physiology, Pharmacology and Neuroscience, University of Bristol Bristol United Kingdom; 5 Department of Psychology, University of Cambridge Cambridge United Kingdom

**Keywords:** point of view, careers in science, research culture, wellbeing, working patterns, caring responsibilities, None

## Abstract

Part-time working can be beneficial for individual academics, and also for academia as a whole. In addition to improving work-life balance and well-being, the benefits of part-time working include increased motivation, reduced burnout, and workplaces that are more diverse and inclusive. Here, six researchers who have experience of working part-time discuss what individuals, employers and funders can do to promote and support part-time working in academia.

Part-time working is relatively rare in academia. However, there are many reasons why some academics might prefer to work part-time. They might have young children or other caring responsibilities, they might have a health condition, or they might undertake additional, non-academic work. In this article, we highlight the benefits of part-time working for academics, discuss the challenges associated with part-time working, and outline what institutions, funding bodies and academics themselves can do to make part-time working a viable option for those who would benefit from it.

The authors of this article all have experience of working part-time, and four of us currently work part-time (see Figure 1 and Box 1). We recently formed a working group to promote the benefits of working part-time, and to make suggestions for ways that employers and funders can support those who want to work part-time. This article is the first output from that group. We acknowledge that our experiences are limited to universities in the UK, and that our decisions to work part-time have primarily been driven by caring responsibilities, so we are keen to expand our group to include more diverse perspectives from across the world; anyone interested in joining the group is asked to contact the corresponding authors by email.

**Figure 1.**
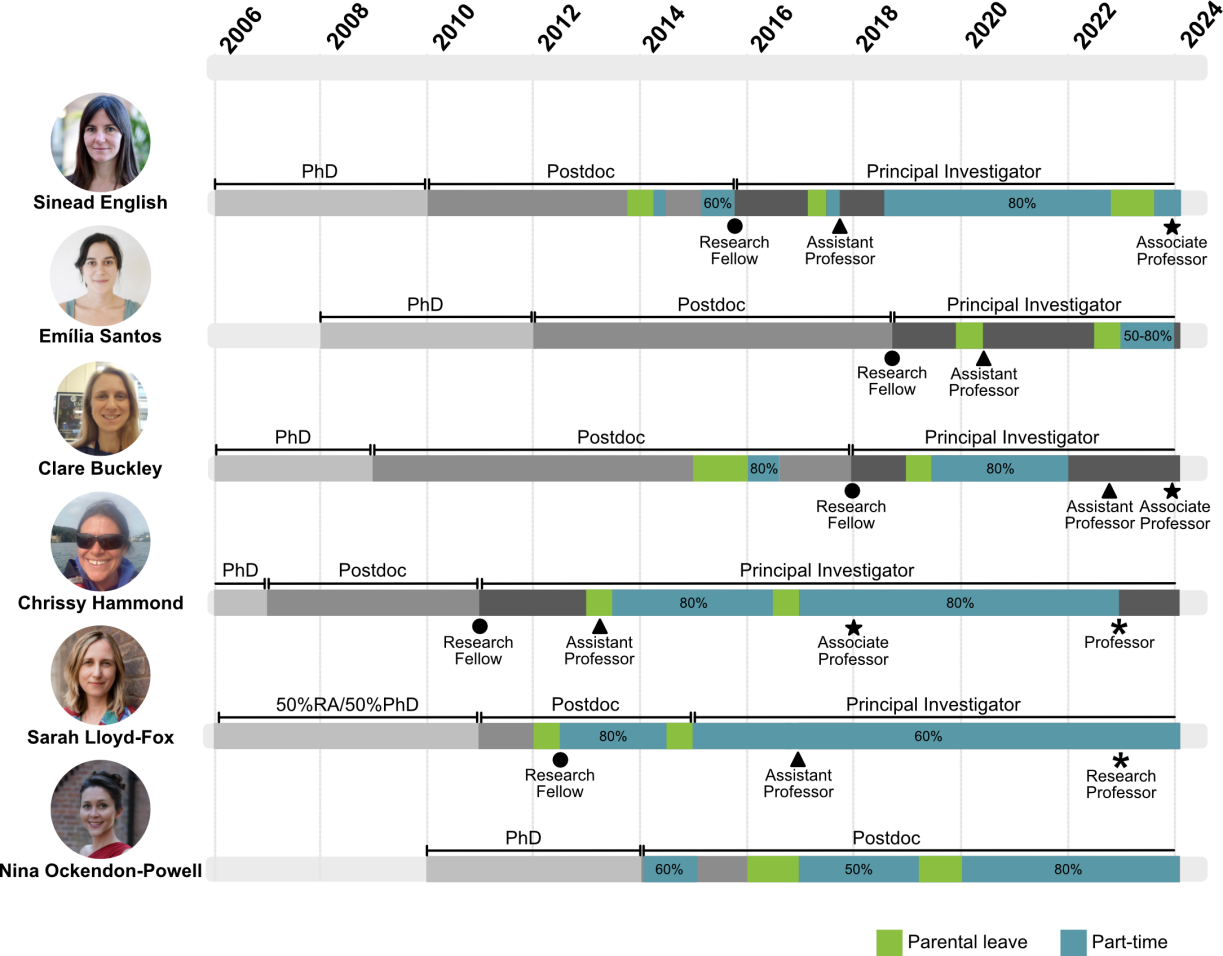
Timelines showing the career paths of the authors from PhD student onwards, including major landmarks such as appointment as a research fellow, assistant professor, associate professor, and professor/research professor. Periods of part-time working (blue), parental leave (green) and full-time working (different shades of grey) are shown (see key). Career paths for the authors.

Box 1. **Different experiences of part-time working.**

We describe our experiences as academic researchers who have worked part-time at some point in their career, ranging from precarious postdoc contracts to professor (Figure 1). While we have written this article as a team, below each author describes their own experience of part-time working.

**Sinead English, associate professor and UKRI Future Leaders Fellow, University of Bristol**

I first worked part-time (0.6 FTE) on returning from maternity leave with my first child: I was on a precarious postdoctoral contract and commuting between cities. I hoped the part-time arrangement would provide some “buffer time” between positions as I juggled new parenting with extra consultancy jobs to pay rent and nursery bills. I went part-time again after securing a permanent position and support in the form of a fellowship for researchers with caring needs (a Dorothy Hodgkin Fellowship from the Royal Society), and have remained 0.8 FTE since. The challenges faced in this role, and a lack of role models and mentors with whom I could discuss these challenges, motivated me to co-create this working group and write this article. Having extra time to spend with my children is rewarding, but I question the implications for career progression, whether my time for research is being squeezed too much, and how to ‘be’ part-time across the diverse facets of academia. Moreover, taking unplanned leave (when, for example, a child is ill) can have a disproportionate impact when you have a shorter working week. I hope that starting a discussion about the positives and negatives of being part-time, and how the latter can be mitigated, will help other researchers to have these conversations in the future.

**M Emília Santos, assistant professor and NERC Independent Research Fellow, University of Cambridge**

I first went part-time after returning from my second maternity leave in 2022 (four months at 0.5 FTE and six months at 0.8 FTE). At the time, it felt impossible to work full-time while caring for my two children and my mental health. However, it was also a struggle to manage my workload while working reduced hours, so I returned to full-time work in 2024. During this time, I reached out to other part-time academics both in person and on social media, asking how they managed workloads and anxiety about not accomplishing enough. This led to connections with many of the authors of this article, and after an initial online meeting, we formed a working group to explore the challenges and benefits of part-time work. These supportive discussions gave me the confidence to return to part-time, and since September 2024, I have been working at 0.8 FTE again. I hope this article will inspire both academics and academic institutions to normalize and promote part-time working patterns in academia.

**Clare Buckley, Sir Henry Dale Research Fellow and senior research fellow, University of Manchester**

I oscillated between working part- and full-time for nine months after returning from maternity leave with my first child, whilst on a postdoctoral contract at King’s College London. At the time I was not able to extend my contract, so I lost pay by going part-time. Following maternity leave with my second child I returned as a new PI at the University of Cambridge to the chaos of the COVID-19 pandemic. I worked part-time for two-and-a-half years (0.8 FTE), which was absolutely necessary to enable me to juggle setting up a new lab with bringing up small children. However, I found it very challenging to manage the academic workload and to secure clear guidance for professional expectations during this time, so returned to full-time working in 2022. I have since moved my research lab to the University of Manchester. I hope that this article will open the narrative to address some of these challenges and to encourage others that part-time workers are a valuable part of the diverse academic workforce.

**Chrissy Hammond, professor, University of Bristol**

I went part-time following the birth of my first child, only returning to full-time after my promotion to professor in 2023. When I had my first child I was a relatively new group leader, and my group was composed of postgraduate research students. At that time, there was no formal parental cover or support for fellows, and as there were no groups working on similar subjects at the university, I needed to stay in touch with my group a lot during my maternity leave, so my husband (not an academic) reduced his hours to 0.8 FTE for three years. While I have loved having the extra time with my children, there is often a frustrating lack of respect for one’s work pattern, with meetings repeatedly scheduled at times clearly marked as out of office. Subsequently, I have pushed for more support for PIs taking long-term leave (both parental and sickness) and for better workload attribution for part-time workers.

**Sarah Lloyd Fox, UKRI Future Leaders Fellow and research professor (principal research associate), University of Cambridge**

Upon writing this I realised it is hard to describe myself as part-time/full-time, as it all depends on how you define ‘work’. I undertook my PhD part time (0.5 FTE) alongside a part-time research assistant post (0.5 FTE) to enable me to finance my education. A brief stint followed as a full-time postdoctoral associate before having my first child. I returned to work at 0.8 FTE, moved to 0.6 FTE when I had a second child, and have remained at 0.6 FTE. During this time, I have become a co-PI, then a PI and been promoted twice. I am also a UKRI Future Leaders Fellow (which comes with an amazing framework of support), though precarity continues and I still do not hold a permanent position after all this time. Over the past decade I have used my part-time role to allow me to spend more time with my children; to undertake non-paid work as a committee member and fundraiser in local childcare facilities, as a leader of extracurricular clubs at schools, as an assistant football coach, and as a trustee of a charity. Working part-time also helps me to work around a chronic health condition that can bring uncertain periods of impact. I have had a wonderful mentor and role model (for part-time work) since my PhD years and have been informed by junior colleagues that I am a role model to them.

**Nina Ockendon-Powell, senior research associate, University of Bristol**

My first part-time role was at 0.6 FTE in my first postdoctoral position – this level was set by European grant funding constraints. I returned to a full-time pattern in my next role (non-academic), until I had my first child and went on maternity leave. I didn’t return to that non-academic job – instead I obtained a 0.5 FTE senior postdoctoral job-share role. This was ideal as it enabled me to transition into working motherhood at a comfortable pace and with peer support. When my child became extremely ill with regular hospitalisations from ages six months to three years, the part-time job share role provided me with the high degree of flexibility I needed to absorb the long absences without impacting performance. Having experimented with differing % hours, and now with two young children, 0.8 FTE feels like a good balance that supports both my family and my career productivity. Having a non-traditional academic career enables me to see where academia can benefit from adopting practices and perspective shifts from other sectors.

## The benefits of part-time working

Research from non-academic sectors has shown that working part-time can help manage stress, reduce burnout, and increase well-being (van Rijswijk et al., 2004; Persson et al., 2022). The flexibility of part-time work, along with longer periods away from the office, can also boost motivation and perspective, resulting in a more positive, efficient, and engaging attitude towards the workplace (Campbell, 2024). Beyond these general benefits, part-time working makes it possible for an individual researcher to make progress in their career while also addressing personal needs outside of research. For example, it enables individuals to spend more time with children or other dependents (Wolfe, 2003), and to develop flexible caring arrangements without the need to work evenings or weekends. Importantly, part-time working can accommodate the academic contributions of those with health conditions that preclude a full-time work schedule.

Additionally, at a time when academic careers are precarious, a shift to part-time working often makes it possible to extend research fellowships or contracts, thus giving breathing space, a modicum of job security, and more time to find the next job. Part-time working can also allow someone to pursue a postgraduate degree without paying tuition fees, or to take on a bridging position in between longer-term, more secure contracts. Alongside caregiving responsibilities and health factors, working part-time also allows academics to diversify their skills and experience, whether through clinical, charitable, private sector, educational or voluntary work, or simply by pursuing a hobby.

Job sharing is a particular approach to part-time working where a full-time post is shared by two people working part-time. In addition to offering the flexibility associated with part-time working, job sharing allows the job holders to offer peer support to each other. Duties are often apportioned within the job share, allowing partners to work to their strengths and do the elements of the job they prefer. Moreover, job-share partners can either split their time across the week so that ‘someone is always on duty’, or they can work similar hours to take advantage of synergies – whatever works best in the setting.

Overall, part-time working patterns can promote a healthy life-work balance and represent a radical departure from the assumption that one must work beyond paid hours to succeed in academia. This shift contributes to the participation of a wider diversity of perspectives and lived experiences, ultimately making academia a more inclusive, diverse, and attractive career option.

## Barriers faced by part-time workers

A major downside of part-time working is that it involves a reduction in salary, and any associated pension. This can mean that it is sometimes only an option for those who can afford it. In some regions, however, high childcare costs can force parents/caregivers to work part-time as this may provide more financial security than working full-time and paying for full-time childcare.

A particular challenge for part-time workers is ensuring that their workload – teaching, research and service – is also reduced accordingly. If this does not happen, an academic working part-time will feel under pressure to deliver the same output as a full-time employee, but in less time and on a lower salary. This is particularly problematic for tasks with fixed deadlines, such as marking exam papers or writing grant applications, since part-time academics have fewer days in which to meet these deadlines. However, many other sectors with hard deadlines manage to enable and support part-time workers and have done so for a long time (Kalleberg, 2000). A possible barrier is that academia as a sector is prone to consider itself differently to others, being somehow less adept at moving with the times and embracing positive changes to working culture.

Another, potentially underappreciated, effect is that the research of a part-time academic suffers disproportionately because it is more ‘squeezable’ than teaching or service. For part-time researchers on fixed-term contracts, the need to do research, to publish papers, and to secure funding to pay their salary – while trying to gain the teaching and administrative experience that is also needed to secure a faculty position – intensifies the sense of pressure and lack of time. It can also be difficult for part-time workers, particularly parents, to participate in the research culture of a department if events like research seminars are scheduled towards the end of the working day, or consistently occur on their non-working day.

Whilst some universities and funders are paving the way when it comes to workload expectations and promotion criteria for part-time workers (see the section on Culture change below), there is still some way to go. Particular problems include slower career progression, increased anxiety due to imposterism, and full-time colleagues giving the impression that they think that part-time workers are less committed or less productive (Wolfe, 2003). Indeed, most of the present authors have been, at some point, discouraged from going part-time by some colleagues.

Meeting the many demands of an academic career is a widely discussed topic (see, for example, Forrester, 2023), with many researchers reporting their normal working week to be 50 hours or more, rather than the 35 or so hours they are contracted to work. So, should a part-time academic working, say, three days a week (i.e., a ‘full-time equivalent’ (FTE) of 60%) be expected to work 30 hours per week (that is, 60% of 50 hours), rather than the 21 hours they are being paid to work? Working such hours would not be possible for many part-time workers, especially those with caring duties and/or those hoping to improve their work-life balance. However, in our collective experience we have found that it is very challenging, verging on impossible, to keep on top of our workloads by following a strict part-time schedule, and there are periods (e.g. funder deadlines, grant review or exam marking) when we have worked late nights or over weekends and family holidays to accommodate these.

A related problem is that some people are worried about going part-time on a temporary basis in case it will not be possible for them to return to full-time working at a later date (when, for example, their children are older) for budgetary reasons.

## Culture change

Given the clear benefits of working part-time, both to the individual and to the institution, we now outline what individual academics, universities and funders can do to encourage and support part-time working patterns.

Existing part-time workers are important both as role models, demonstrating that successful part-time work is possible and that part-time workers can be promoted, and as mentors, advising individuals considering part-time working and helping them to adjust. Moreover, in our experience (Box 1), role models create open discourse about work-life balance and the barriers and benefits to part-time working.

Such a dialogue can benefit full-time workers too: transparency and open communication about when they take time off for family events or for their own enjoyment helps to break down the assumption in academia that to succeed requires ‘all the hours all the time’. When this is embraced and modeled by senior colleagues, it creates a better research culture for everyone, where the implicit message is that working in a pattern that suits your life circumstances is not a barrier to success. Even small signaling actions, adoptable by everyone, can help drive cultural change, such as email signatures that make explicit no response is required outside the recipient’s work pattern.

While some institutions have made progress in encouraging and supporting part-time working, many have not. Here are five changes we would like to see embraced universally. First, the use of workload modeling to proactively and fairly divide, assign and acknowledge work proportional to FTE, including traditional tasks such as lecturing and marking, but also hidden service tasks that benefit research culture, such as mentoring, career support and actions towards better equality, diversity and inclusion.

Second, ensuring that policies about promotion include a transparent mechanism for taking account of periods of part-time work and career breaks, and also take account of contributions to leadership, civic actions and research culture alongside traditional factors (such as research outputs, funding and impact).

Third, conducting regular surveys of research culture to highlight approaches that are working well, and those that need improvement. Fourth, providing career progression support for part-time workers: for example, line managers could discuss promotion pathways on an annual basis, and senior colleagues could explore opportunities for ‘reverse mentorship’ to better understand how part-time academics can be successful. Fifth, more can be done to celebrate the value that part-time academics bring to research culture at institutional, national and international levels, including insights gained from caregiving, charity, community, and clinical roles.

Finally, there are two actions that we encourage funders to take. First, include explicit wording in funding calls to make it clear that grant-holders can work part-time. Second, continue and expand the use of narrative CVs, and ensure that panels assessing candidates for fellowships, jobs and promotions properly take account of part-time working and career breaks so that institutions can benefit from the rich tapestry of experiences that part-time academics can bring.

Widespread adoption of these relatively simple and largely cost-neutral policies has the potential to benefit a large number of individual academics and the academic sector as a whole.

## Data Availability

There are no data associated with this article.
